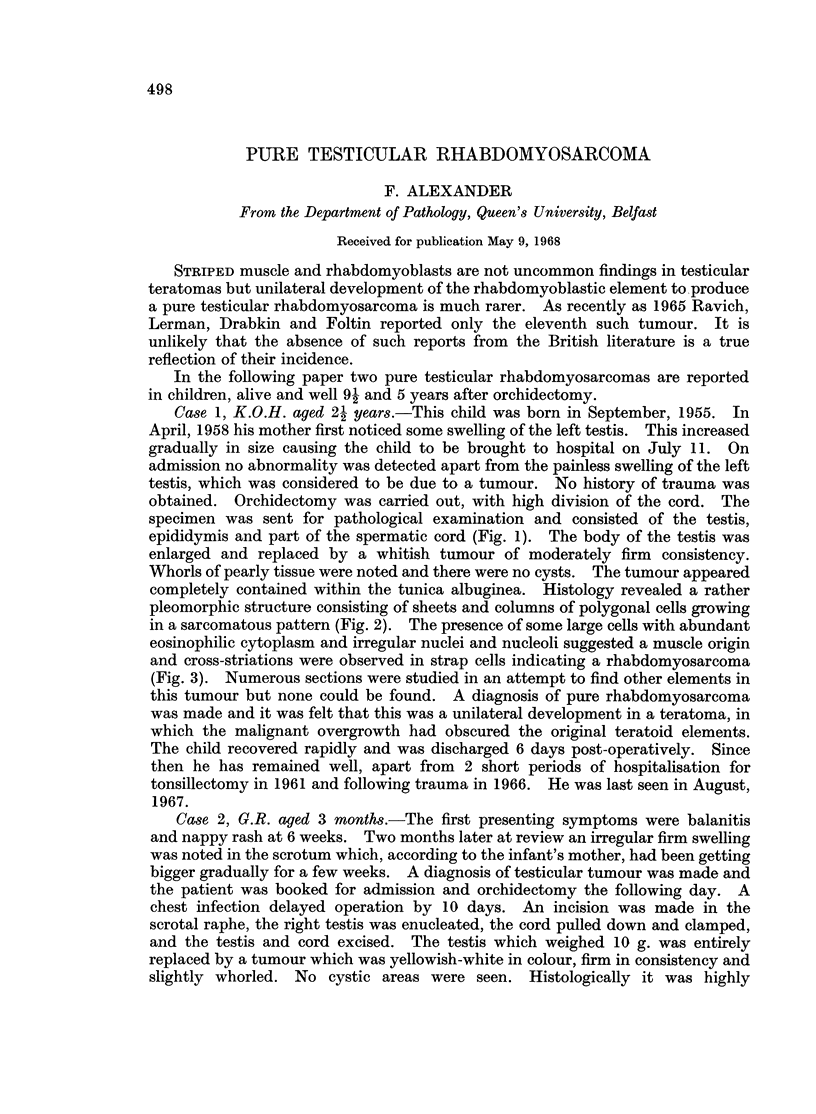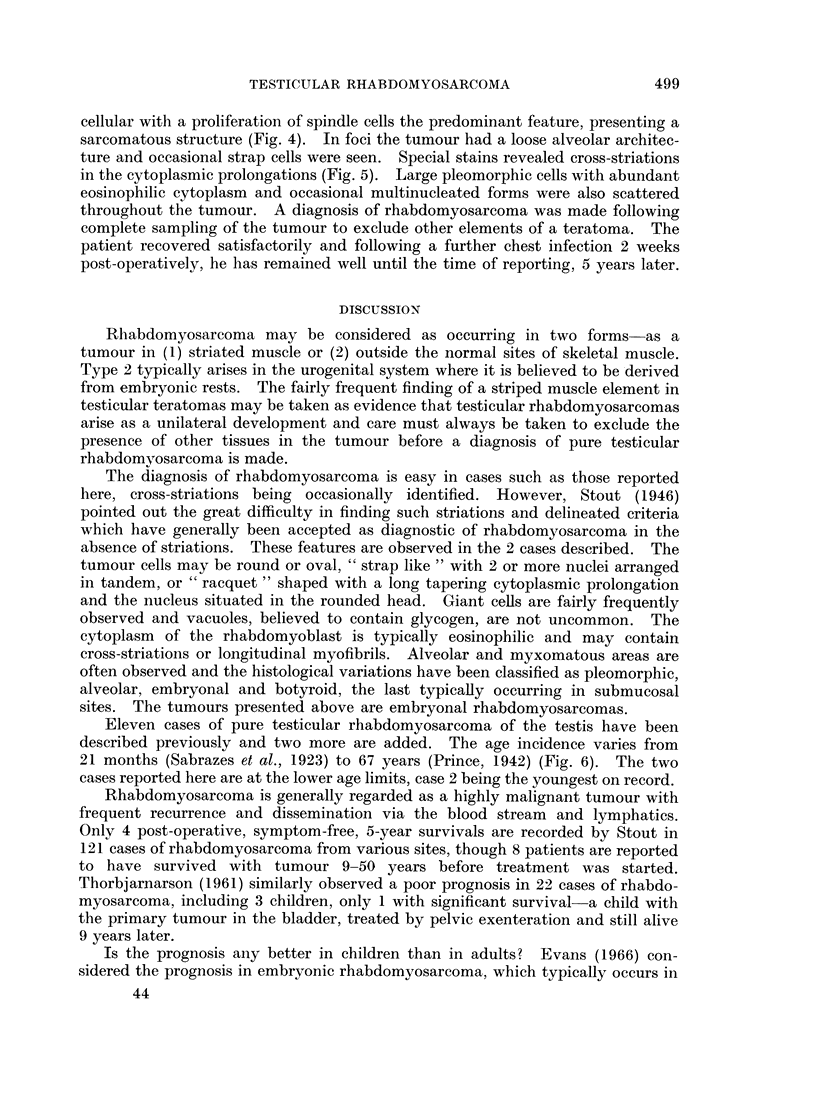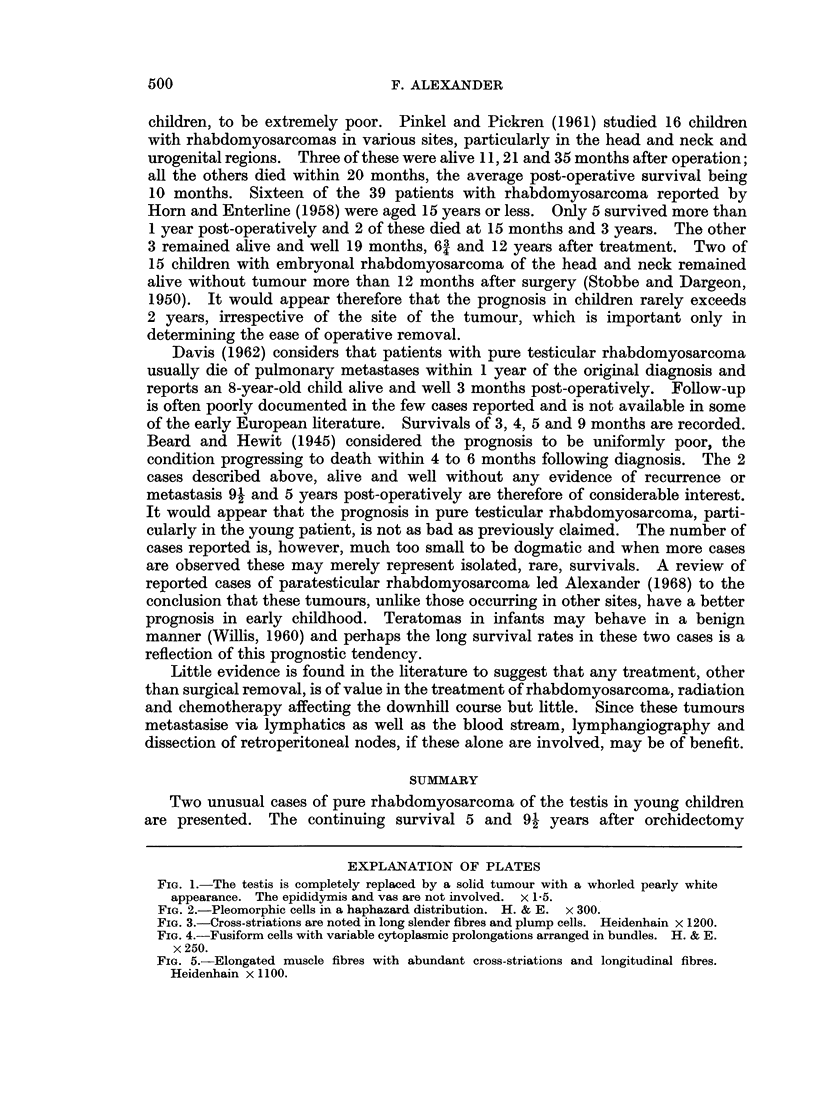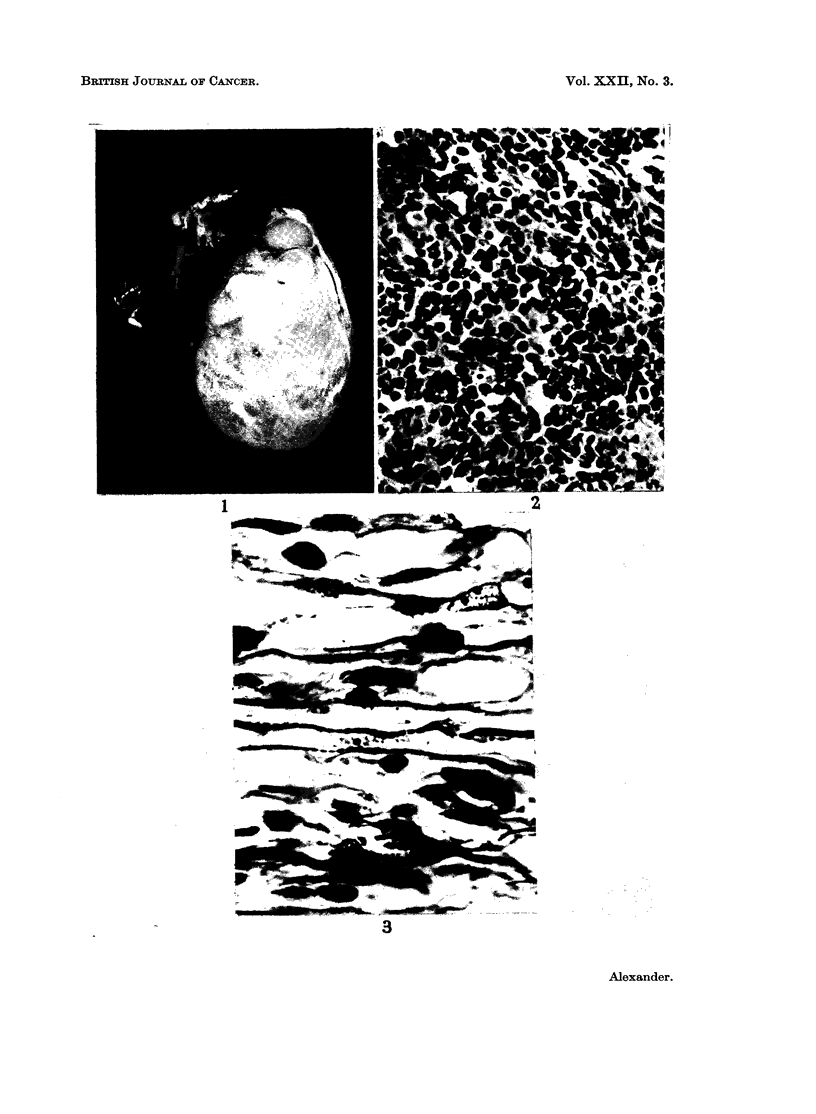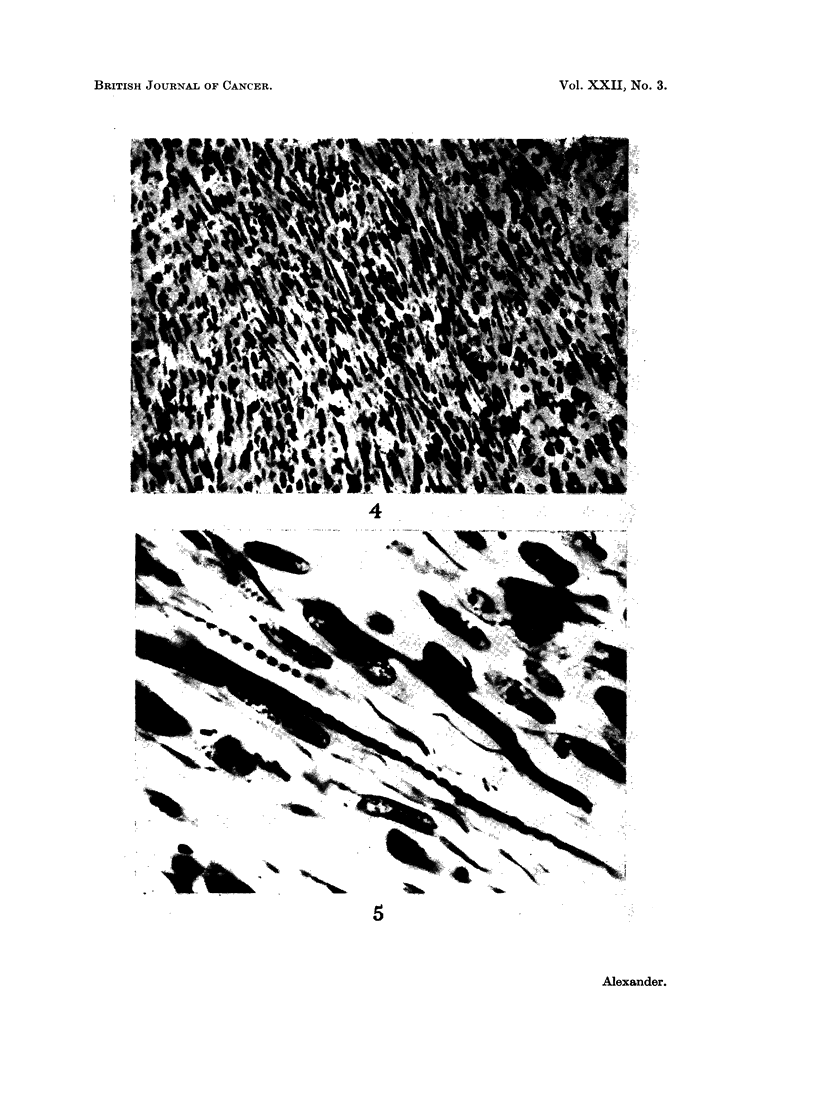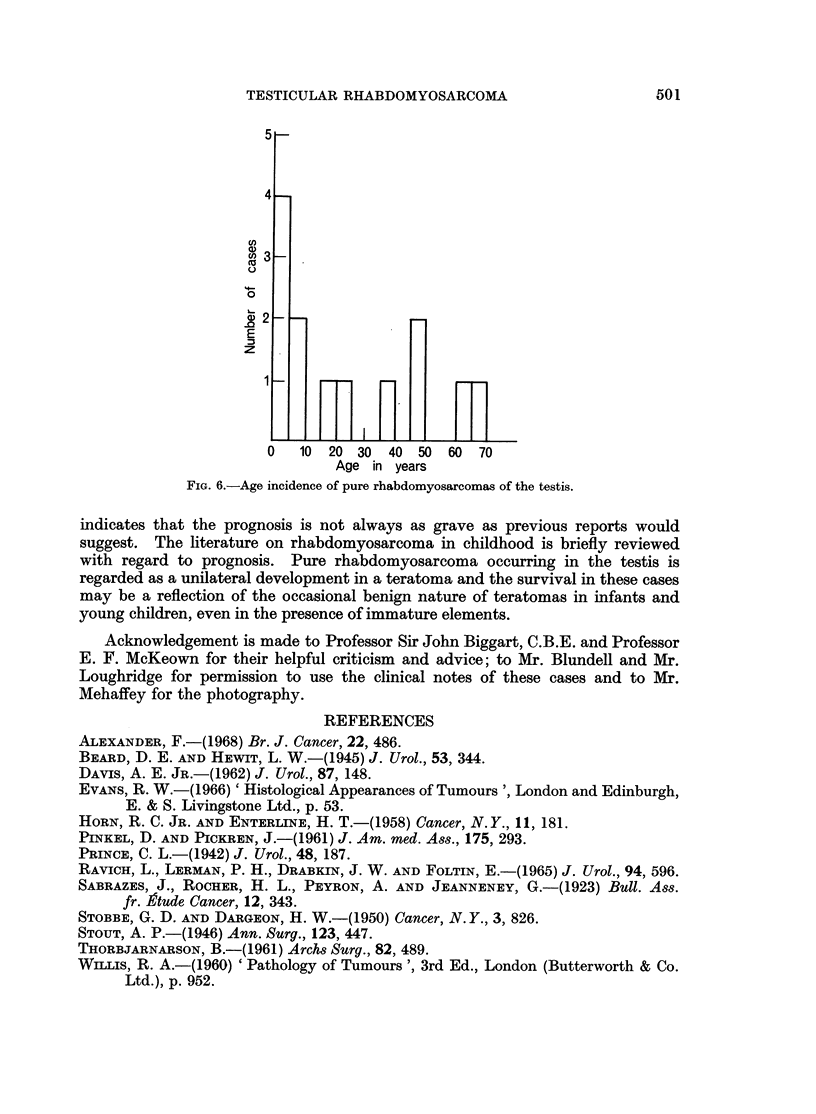# Pure testicular rhabdomyosarcoma.

**DOI:** 10.1038/bjc.1968.58

**Published:** 1968-09

**Authors:** F. Alexander

## Abstract

**Images:**


### PURE TESTICULAR RHABDOMYOSARCOMA

F. ALEXANDER

*From the Department of Pathology, Queen's University, Belfast*

Received for publication May 9, 1968

STRIPED muscle and rhabdomyoblasts are not uncommon findings in testicular teratomas but unilateral development of the rhabdomyoblastic element to produce a pure testicular rhabdomyosarcoma is much rarer. As recently as 1965 Ravich, Lerman, Drabkin and Foltin reported only the eleventh such tumour. It is unlikely that the absence of such reports from the British literature is a true reflection of their incidence.

In the following paper two pure testicular rhabdomyosarcomas are reported in children, alive and well 9½ and 5 years after orchidectomy.

*Case* 1, *K.O.H. aged* 2½ *years*.—This child was born in September, 1955. In April, 1958 his mother first noticed some swelling of the left testis. This increased gradually in size causing the child to be brought to hospital on July 11. On admission no abnormality was detected apart from the painless swelling of the left testis, which was considered to be due to a tumour. No history of trauma was obtained. Orchidectomy was carried out, with high division of the cord. The specimen was sent for pathological examination and consisted of the testis, epididymis and part of the spermatic cord (Fig. 1). The body of the testis was enlarged and replaced by a whitish tumour of moderately firm consistency. Whorls of pearly tissue were noted and there were no cysts. The tumour appeared completely contained within the tunica albuginea. Histology revealed a rather pleomorphic structure consisting of sheets and columns of polygonal cells growing in a sarcomatous pattern (Fig. 2). The presence of some large cells with abundant eosinophilic cytoplasm and irregular nuclei and nucleoli suggested a muscle origin and cross-striations were observed in strap cells indicating a rhabdomyosarcoma (Fig. 3). Numerous sections were studied in an attempt to find other elements in this tumour but none could be found. A diagnosis of pure rhabdomyosarcoma was made and it was felt that this was a unilateral development in a teratoma, in which the malignant overgrowth had obscured the original teratoid elements. The child recovered rapidly and was discharged 6 days post-operatively. Since then he has remained well, apart from 2 short periods of hospitalisation for tonsillectomy in 1961 and following trauma in 1966. He was last seen in August, 1967.

*Case* 2, *G.R. aged* 3 *months*.—The first presenting symptoms were balanitis and nappy rash at 6 weeks. Two months later at review an irregular firm swelling was noted in the scrotum which, according to the infant's mother, had been getting bigger gradually for a few weeks. A diagnosis of testicular tumour was made and the patient was booked for admission and orchidectomy the following day. A chest infection delayed operation by 10 days. An incision was made in the scrotal raphe, the right testis was enucleated, the cord pulled down and clamped, and the testis and cord excised. The testis which weighed 10 g. was entirely replaced by a tumour which was yellowish-white in colour, firm in consistency and slightly whorled. No cystic areas were seen. Histologically it was highly cellular with a proliferation of spindle cells the predominant feature, presenting a sarcomatous structure (Fig. 4). In foci the tumour had a loose alveolar architecture and occasional strap cells were seen. Special stains revealed cross-striations in the cytoplasmic prolongations (Fig. 5). Large pleomorphic cells with abundant eosinophilic cytoplasm and occasional multinucleated forms were also scattered throughout the tumour. A diagnosis of rhabdomyosarcoma was made following complete sampling of the tumour to exclude other elements of a teratoma. The patient recovered satisfactorily and following a further chest infection 2 weeks post-operatively, he has remained well until the time of reporting, 5 years later.

### DISCUSSION

Rhabdomyosarcoma may be considered as occurring in two forms—as a tumour in (1) striated muscle or (2) outside the normal sites of skeletal muscle. Type 2 typically arises in the urogenital system where it is believed to be derived from embryonic rests. The fairly frequent finding of a striped muscle element in testicular teratomas may be taken as evidence that testicular rhabdomyosarcomas arise as a unilateral development and care must always be taken to exclude the presence of other tissues in the tumour before a diagnosis of pure testicular rhabdomyosarcoma is made.

The diagnosis of rhabdomyosarcoma is easy in cases such as those reported here, cross-striations being occasionally identified. However, Stout (1946) pointed out the great difficulty in finding such striations and delineated criteria which have generally been accepted as diagnostic of rhabdomyosarcoma in the absence of striations. These features are observed in the 2 cases described. The tumour cells may be round or oval, " strap like " with 2 or more nuclei arranged in tandem, or " racquet " shaped with a long tapering cytoplasmic prolongation and the nucleus situated in the rounded head. Giant cells are fairly frequently observed and vacuoles, believed to contain glycogen, are not uncommon. The cytoplasm of the rhabdomyoblast is typically eosinophilic and may contain cross-striations or longitudinal myofibrils. Alveolar and myxomatous areas are often observed and the histological variations have been classified as pleomorphic, alveolar, embryonal and botyroid, the last typically occurring in submucosal sites. The tumours presented above are embryonal rhabdomyosarcomas.

Eleven cases of pure testicular rhabdomyosarcoma of the testis have been described previously and two more are added. The age incidence varies from 21 months (Sabrazes *et al.*, 1923) to 67 years (Prince, 1942) (Fig. 6). The two cases reported here are at the lower age limits, case 2 being the youngest on record.

Rhabdomyosarcoma is generally regarded as a highly malignant tumour with frequent recurrence and dissemination via the blood stream and lymphatics. Only 4 post-operative, symptom-free, 5-year survivals are recorded by Stout in 121 cases of rhabdomyosarcoma from various sites, though 8 patients are reported to have survived with tumour 9–50 years before treatment was started. Thorbjarnarson (1961) similarly observed a poor prognosis in 22 cases of rhabdomyosarcoma, including 3 children, only 1 with significant survival—a child with the primary tumour in the bladder, treated by pelvic exenteration and still alive 9 years later.

Is the prognosis any better in children than in adults? Evans (1966) considered the prognosis in embryonic rhabdomyosarcoma, which typically occurs in children, to be extremely poor. Pinkel and Pickren (1961) studied 16 children with rhabdomyosarcomas in various sites, particularly in the head and neck and urogenital regions. Three of these were alive 11, 21 and 35 months after operation; all the others died within 20 months, the average post-operative survival being 10 months. Sixteen of the 39 patients with rhabdomyosarcoma reported by Horn and Enterline (1958) were aged 15 years or less. Only 5 survived more than 1 year post-operatively and 2 of these died at 15 months and 3 years. The other 3 remained alive and well 19 months, 6¾ and 12 years after treatment. Two of 15 children with embryonal rhabdomyosarcoma of the head and neck remained alive without tumour more than 12 months after surgery (Stobbe and Dargeon, 1950). It would appear therefore that the prognosis in children rarely exceeds 2 years, irrespective of the site of the tumour, which is important only in determining the ease of operative removal.

Davis (1962) considers that patients with pure testicular rhabdomyosarcoma usually die of pulmonary metastases within 1 year of the original diagnosis and reports an 8-year-old child alive and well 3 months post-operatively. Follow-up is often poorly documented in the few cases reported and is not available in some of the early European literature. Survivals of 3, 4, 5 and 9 months are recorded. Beard and Hewit (1945) considered the prognosis to be uniformly poor, the condition progressing to death within 4 to 6 months following diagnosis. The 2 cases described above, alive and well without any evidence of recurrence or metastasis 9½ and 5 years post-operatively are therefore of considerable interest. It would appear that the prognosis in pure testicular rhabdomyosarcoma, particularly in the young patient, is not as bad as previously claimed. The number of cases reported is, however, much too small to be dogmatic and when more cases are observed these may merely represent isolated, rare, survivals. A review of reported cases of paratesticular rhabdomyosarcoma led Alexander (1968) to the conclusion that these tumours, unlike those occurring in other sites, have a better prognosis in early childhood. Teratomas in infants may behave in a benign manner (Willis, 1960) and perhaps the long survival rates in these two cases is a reflection of this prognostic tendency.

Little evidence is found in the literature to suggest that any treatment, other than surgical removal, is of value in the treatment of rhabdomyosarcoma, radiation and chemotherapy affecting the downhill course but little. Since these tumours metastasise via lymphatics as well as the blood stream, lymphangiography and dissection of retroperitoneal nodes, if these alone are involved, may be of benefit.

### SUMMARY

Two unusual cases of pure rhabdomyosarcoma of the testis in young children are presented. The continuing survival 5 and 9½ years after orchidectomy

---

### EXPLANATION OF PLATES

FIG. 1.—The testis is completely replaced by a solid tumour with a whorled pearly white appearance. The epididymis and vas are not involved. × 1·5.

FIG. 2.—Pleomorphic cells in a haphazard distribution. H. & E. × 300.

FIG. 3.—Cross-striations are noted in long slender fibres and plump cells. Heidenhain × 1200.

FIG. 4.—Fusiform cells with variable cytoplasmic prolongations arranged in bundles. H. & E. × 250.

FIG. 5.—Elongated muscle fibres with abundant cross-striations and longitudinal fibres. Heidenhain × 1100.

1

2

3

Alexander.

4

5

Alexander.

FIG. 6.—Age incidence of pure rhabdomyosarcomas of the testis.

indicates that the prognosis is not always as grave as previous reports would suggest. The literature on rhabdomyosarcoma in childhood is briefly reviewed with regard to prognosis. Pure rhabdomyosarcoma occurring in the testis is regarded as a unilateral development in a teratoma and the survival in these cases may be a reflection of the occasional benign nature of teratomas in infants and young children, even in the presence of immature elements.

Acknowledgement is made to Professor Sir John Biggart, C.B.E. and Professor E. F. McKeown for their helpful criticism and advice; to Mr. Blundell and Mr. Loughridge for permission to use the clinical notes of these cases and to Mr. Mehaffey for the photography.

### REFERENCES

ALEXANDER, F.—(1968) *Br. J. Cancer*, **22**, 486.
BEARD, D. E. AND HEWIT, L. W.—(1945) *J. Urol.*, **53**, 344.
DAVIS, A. E. JR.—(1962) *J. Urol.*, **87**, 148.
EVANS, R. W.—(1966) ‘Histological Appearances of Tumours’, London and Edinburgh, E. & S. Livingstone Ltd., p. 53.
HORN, R. C. JR. AND ENTERLINE, H. T.—(1958) *Cancer, N.Y.*, **11**, 181.
PINKEL, D. AND PICKREN, J.—(1961) *J. Am. med. Ass.*, **175**, 293.
PRINCE, C. L.—(1942) *J. Urol.*, **48**, 187.
RAVICH, L., LERMAN, P. H., DRABKIN, J. W. AND FOLTIN, E.—(1965) *J. Urol.*, **94**, 596.
SABRAZES, J., ROCHER, H. L., PEYRON, A. AND JEANNENEY, G.—(1923) *Bull. Ass. fr. Étude Cancer*, **12**, 343.
STOBBE, G. D. AND DARGEON, H. W.—(1950) *Cancer, N.Y.*, **3**, 826.
STOUT, A. P.—(1946) *Ann. Surg.*, **123**, 447.
THORBJARNARSON, B.—(1961) *Archs Surg.*, **82**, 489.
WILLIS, R. A.—(1960) ‘Pathology of Tumours’, 3rd Ed., London (Butterworth & Co. Ltd.), p. 952.